# LRP1 Controls cPLA_2_ Phosphorylation, ABCA1 Expression and Cellular Cholesterol Export

**DOI:** 10.1371/journal.pone.0006853

**Published:** 2009-08-31

**Authors:** Li Zhou, Hong Y. Choi, Wei-Ping Li, Fang Xu, Joachim Herz

**Affiliations:** 1 Department of Molecular Genetics, UT Southwestern Medical Center, Dallas, Texas, United States of America; 2 Department of Cell Biology, UT Southwestern Medical Center, Dallas, Texas, United States of America; 3 Department of Human Nutrition, UT Southwestern Medical Center, Dallas, Texas, United States of America; Leiden University Medical Center, Netherlands

## Abstract

**Background:**

ATP-binding cassette transporter A1 mediates apolipoprotein AI-dependent efflux of cholesterol and thereby removes cholesterol from peripheral tissues. ABCA1 expression is tightly regulated and deficiency of this cholesterol transporter results in cholesterol accumulation within cells. Low-density lipoprotein receptor-related protein 1 (LRP1) participates in lipid metabolism and energy homeostasis by endocytosis of apolipoprotein E-containing lipoproteins and modulation of cellular proliferation signals.

**Methods and Principal Findings:**

In the present study, we demonstrate a new role for LRP1 in reverse cholesterol transport. Absence of LRP1 expression results in increased PDGFRβ signaling and sequential activation of the mitogen-activated protein kinase signaling pathway, which increases phosphorylation of cytosolic phospholipase A_2_ (cPLA_2_). Phosphorylated and activated cPLA_2_ releases arachidonic acid from the phospholipid pool. Overproduction of arachidonic acid suppresses the activation of LXR/RXR heterodimers bound to the promoter of LXR regulated genes such as ABCA1, resulting in greatly reduced ABCA1 expression.

**Conclusions and Significance:**

LRP1 regulates LXR-mediated gene transcription and participates in reverse cholesterol transport by controlling cPLA_2_ activation and ABCA1 expression. LRP1 thus functions as a physiological integrator of cellular lipid homeostasis with signals that regulate cellular proliferation and vascular wall integrity.

## Introduction

Cholesterol is an essential component of cell membrane and necessary for normal cellular function, including cell proliferation [1]. Excess cholesterol accumulation, however, can result in pathological consequences. This is particularly true for cells of the arterial wall, where accumulation of cholesterol initiates atherosclerosis [2], [3]. A complex homeostatic network has therefore evolved to modulate cholesterol biosynthesis, transport and excretion. Studies on Tangier disease have revealed an important role of ATP-binding cassette transporter A1 (ABCA1) in cholesterol homeostasis [4], [5], [6]. As a membrane transporter, ABCA1 facilitates the formation of HDL via apolipoprotein AI (apoAI)-mediated efflux of cholesterol and phospholipids from many tissues [7], [8], [9]. This constitutes the initial step of reverse cholesterol transport, and ultimately leads to the elimination of cholesterol from the body [10], [11], [12]. Functional defects in the ABCA1 protein that impair its ability to mediate cellular cholesterol efflux can thus result in deposition of cholesterol within the tissues.

As a member of the LDL receptor (LDLR) family, LDL receptor-related protein 1 (LRP1) was initially identified as a cellular receptor that endocytoses apolipoprotein E (apoE)-enriched lipoproteins [13], [14], [15], [16]. Subsequent studies have shown, however, that LRP1 is a highly multifunctional receptor that not only mediates the endocytosis of a broad spectrum of macromolecules, but also functions as a modulator and integrator of several fundamental cell signaling pathways [17], [18], [19], [20]. One of these involves signaling by platelet-derived growth factor BB (PDGF-BB).

LRP1 forms a complex with the PDGF receptor β (PDGFRβ) in clathrin-coated pits and caveolae [17], [21], [22]. Absence of LRP1 in vascular smooth muscle cells in the mouse (smLRP1−/−) leads to increased PDGFRβ expression, greatly accelerated development of atherosclerotic lesions, and prominent accumulation of cholesterol in the vessel wall [18]. LRP1 also regulates Wnt5a signaling during adipocyte differentiation and thereby serves as an endogenous regulator of cellular cholesterol and triglyceride homeostasis [20].

Although LRP1 and ABCA1 therefore both play import ant and distinct roles in cellular cholesterol homeostasis and atherosclerosis, the functional interaction between these two membrane proteins has never been investigated. The accumulation of cholesterol in the vascular wall of smLRP−/− mice, even in the presence of normal or only moderately increased plasma cholesterol levels, and in particular the massive accumulation that occurs in the absence of the LDL receptor suggested a disruption of cholesterol export from the LRP1-deficient smooth muscle cells as a potential underlying mechanism. In the present study, we have taken advantage of the smLRP1−/− mice to investigate the consequences of LRP1 deficiency for ABCA1 expression and function *in vitro* and *in vivo*. Our goal was to investigate if and how LRP1 regulates ABCA1 functional expression and thereby cholesterol efflux in the vascular wall.

## Results

### SmLRP1−/− mice have increased total cholesterol levels in the aorta

**Figure 1:**
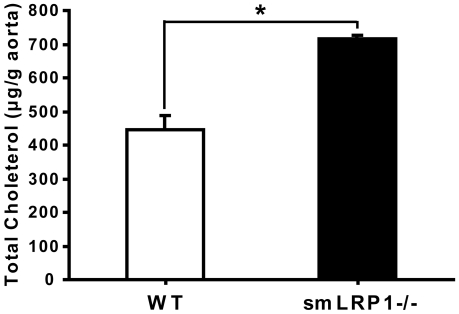
GC/MS analysis of cholesterol in the mouse aorta. The aorta from the aortic root to the iliac bifurcation was isolated, and the surrounding connective tissue was carefully removed under a dissecting microscope. The aorta was weighed after superficial drying with tissue paper. Cholesterol in the aorta was determined by GC/MS and the amount of total cholesterol (µg) was normalized by the weight of the aorta (g). Significantly increased total cholesterol levels were found in the aorta of the smLRP1−/− mice (black bar). 4-month old female littermates of the indicated genotypes were used for the depicted experiment. LRP^flox/flox^ animals lacking the Sm22Cre transgene are indistinguishable from wild type [18] and were used as control groups. Equivalent results were obtained for male mice and throughout the lifetime of the animals (Figure S1). Values were presented as mean±S.E.M. *p<0.05 (compared to wild type, n = 3/group).

LRP1 is a multifunctional endocytic receptor participating in the removal of TG-rich VLDL and chylomicron remnants in the liver [23]. Recent studies have shown that LRP1 is also involved in cholesterol storage and fatty acid synthesis in fibroblasts and adipocytes [20], [24]. However, cholesterol levels in the aortas of young smLRP1−/− mice in the absence of atherosclerotic lesions have so far not been investigated. Using gas chromatography and mass spectrometry (GC/MS), we found a significant increase in total cholesterol in the aortas of smLRP1−/− mice (Figure 1 and Figure S1). Only aortas lacking any morphologically discernible atherosclerotic lesions or plaques were analyzed. These increased cholesterol levels are thus not caused by the presence of cholesterol in plaques or advanced lesions, but reflect either increased lipoprotein uptake or the inability of LRP1-deficient smooth muscle cells to export endogenous cholesterol in the presence of an intact endothelium.

### Lack of LRP1 expression in SMCs results in reduced ABCA1 protein expression in the aorta

**Figure 2:**
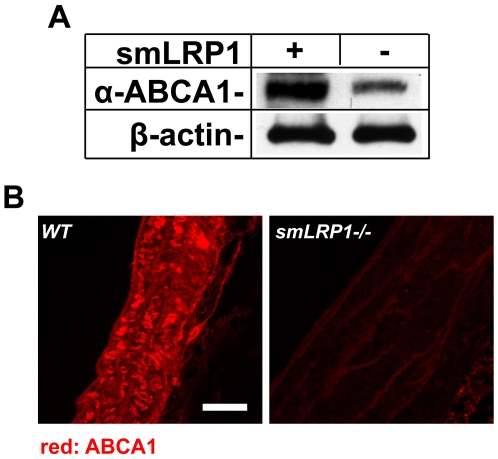
Detection of ABCA1 protein expression in the aorta by Western blotting and immunofluorescence microscopy. *A*. Aortas of the indicated genotypes were homogenized in lysis buffer. 10 µg of protein extracts were subjected to 4–15% SDS-PAGE gel, and the blot was then probed with a rat anti-mouse ABCA1 monoclonal antibody. β-actin was detected to demonstrate equal loading. Three independent experiments were quantitated. ABCA1 protein expression in smLRP- aortas was reduced to 0.39±0.04 compared to wild type. *B*. After fixation, aortas were embedded in paraffin and 5 µm sections were prepared. WT (left) and smLRP1−/− (right) aorta sections were then stained with the rat anti-mouse ABCA1 monoclonal antibody, and detected by Alexa-Fluor 568-conjugated goat anti-rat IgG (red). Images were acquired on a Leica TCS SP confocal microscope. Scale bar: 25 µm. Both assays showed greatly reduced ABCA1 protein expression in the aorta of the smLRP1−/− mice.

Because ABCA1 plays an important role in lipid transport [25], impaired expression of ABCA1 in the aorta could potentially be responsible for the increased total cholesterol and the enhanced sensitivity to atherosclerosis in the smLRP1−/− mice. To examine the expression of ABCA1 protein in wild type and smLRP1−/− mice, we immunoblotted and immunostained the aorta with a specific monoclonal rat anti-mouse ABCA1 antibody. As shown in Figure 2A, a significant reduction in ABCA1 protein in the smLRP1−/− mice was revealed by Western blotting. This was supported by immunohistochemical staining of aorta sections, which also showed greatly diminished ABCA1 protein in the smLRP1−/− aortas (Figure 2B).

### Increased cellular lipid accumulation is detected in LRP1-deficient SMCs

**Figure 3:**
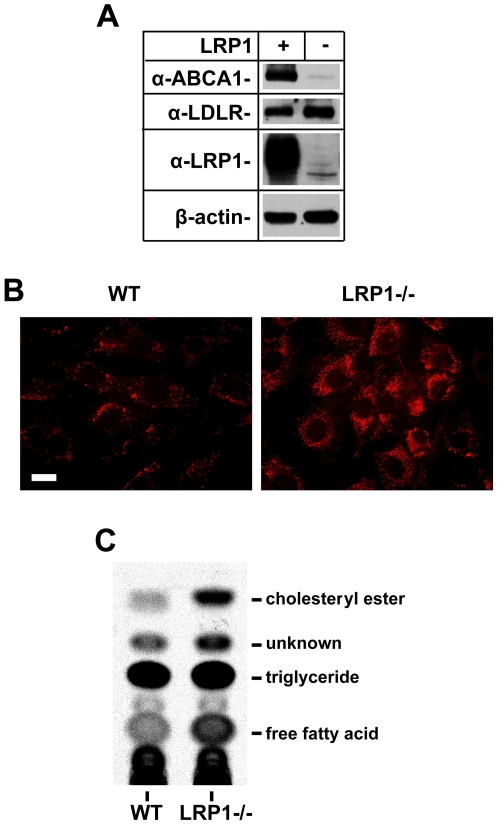
Lipid analysis of primary SMCs. *A*. Primary SMCs of the indicated genotypes were lysed in freshly prepared lysis buffer. 10 µg of protein extracts were loaded on 4–15% SDS-PAGE gels. Blots were then probed with the rat anti-mouse ABCA1 monoclonal antibody, a rabbit anti-LDLR polyclonal antibody, and a rabbit anti-LRP1 polyclonal antibody. β-actin was detected to demonstrate equal loading. Western blotting consistently confirmed significantly reduced (∼5 fold) ABCA1 and increased LDLR protein expression (1.67±0.12, n = 3 and [36]) in primary SMCs lacking LRP1. *B*. Cells grown on coverslips were fixed with 10% formalin and stained with 4 mg/ml Oil Red O in isopropyl alcohol. Oil red O staining showed increased lipid accumulation in LRP1-deficient cells (right panel). Images were captured on a Zeiss fluorescence microscope. Scale bar: 12.5 µm. *C*. Primary SMCs of the indicated genotypes were harvested and lysed after incubation with ^14^C-labelled oleic acid. Lipids were extracted with chloroform/methanol (2∶1, v∶v) and processed for thin layer chromatography (TLC) analysis. The TLC plates were exposed to a phosphor imaging plate. Cholesteryl ester, triglycerides and free fatty acids in the lipid extracts were separated in a solvent system consisting of hexane/ethyl ether/acetic acid (80∶20∶1, v∶v∶v). ^3^H-labelled cholesterol recovery solution containing cholesteryl oleate ester, triglyceride and oleate was used as lipid standards. Increased amounts of cholesteryl ester and free fatty acids were observed in LRP1−/− SMCs.

To characterize the changes of cellular lipids in the presence and absence of LRP1, primary SMCs were generated from the mouse aorta. First, we confirmed by immunoblotting that ABCA1 protein levels are also significantly reduced in primary SMCs lacking LRP1 expression (Figure 3A). We further detected an increase in LDLR expression in the absence of LRP1. Next, we stained the cells with Oil Red O to visualize neutral lipid deposits. Oil Red O staining showed increased lipid accumulation in the LRP1-deficient cells (Figure 3B, right panel). To further analyze the components of increased lipids in the LRP1−/− SMCs, we extracted lipids four hours after cellular uptake of ^14^C-labelled oleic acid and separated the lipid extracts by thin layer chromatography (TLC). We found increased amounts of cholesterol ester and free fatty acids (Figure 3C) in the LRP1-deficient SMCs. These data suggested that LRP1 could potentially regulate cellular lipid trafficking by controlling ABCA1 protein expression.

### LRP1 regulates ABCA1 expression at the transcriptional level

**Figure 4:**
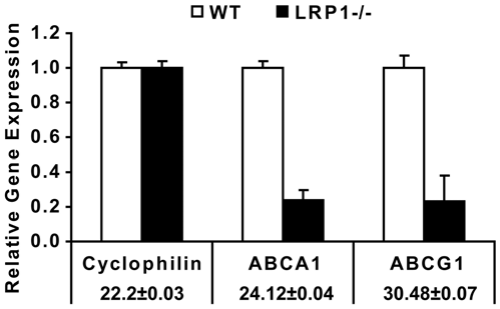
Real-time PCR quantification of ABCA1 and ABCG1 mRNA in primary SMCs. 2 µg of total RNA from SMCs of the indicated genotypes were prepared, and processed for real-time PCR. PCR reactions were performed in triplicate. The relative amount of mRNA was calculated using the comparative threshold cycle method. Mouse cyclophilin mRNA was used as the invariant control. ABCA1 and ABCG1 mRNA levels are significantly diminished in LRP1-deficient SMCs (black bar). Wild type *C_T_* values are shown±S.D. Assays were performed in triplicate, standard deviations are shown.

To detect whether the reduced ABCA1 protein levels are caused by decreased gene transcription, we next quantitated the mRNA levels of ABCA1 and ATP-binding cassette transporter G1 (ABCG1), another transporter protein involved in cholesterol efflux, both in WT and LRP1−/− SMCs using real-time PCR. The result showed abundant expression of ABCA1 in primary SMCs, whereas transcription of ABCG1 was almost undetectable (Figure 4). Moreover, we noted a reduction of approximately 80% in ABCA1 mRNA expression in the LRP1-deficient SMCs. This robust decrease of ABCA1 mRNA is consistent with the significant reduction of ABCA1 protein, suggesting that in the absence of LRP1, ABCA1 expression is repressed at the transcriptional level.

### Transcription of LXRs and RXRs is not suppressed in the LRP-deficient SMCs

**Figure 5:**
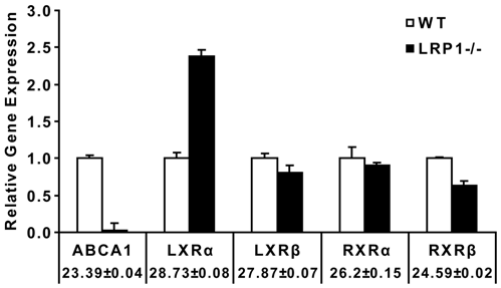
Quantification of LXR and RXR mRNA by real-time PCR in primary SMCs. 2 µg of total RNA from WT and LRP1−/− SMCs were prepared and subjected to real-time PCR quantification. Relative expression ratios represents the amount of mRNA in the LRP1−/− SMCs relative to that in the wild type cells, which was arbitrarily defined as 1. The number underneath each gene represented the wild type C*_T_* values±S.D. mRNA levels of the transcriptional regulators of ABCA1, namely LXRs and RXRs, in the LRP1−/− SMCs (black bars) were comparable to those in the wild type cells (white bars). Assays were performed in triplicate, standard deviations are shown.

Liver X receptors (LXRs) and retinoid X receptors (RXRs) are the key transcriptional regulators of ABCA1 [25], [26]. LXRs form obligate heterodimers with the RXRs and the heterodimers bind to the LXR-responsive elements (LXREs) in the proximal promoter region of ABCA1. Thus, decreased LXR and RXR gene transcription could be responsible for the reduction in ABCA1 mRNA levels. To examine the mRNA levels of LXRs and RXRs in the WT and LRP1−/− SMCs, we performed real-time PCR. Our results showed that the LXR and RXR mRNA levels in the LRP1-deficient SMCs were comparable to those present in the wild type cells and LXRα levels were even increased 2.4 fold (Figure 5), suggesting that the reduced ABCA1 expression in the LRP1−/− SMCs is not due to changes in gene transcription of LXRs and RXRs.

### Repressed ABCA1 transcription in the absence of LRP1 is reversed by LXR agonists

**Figure 6:**
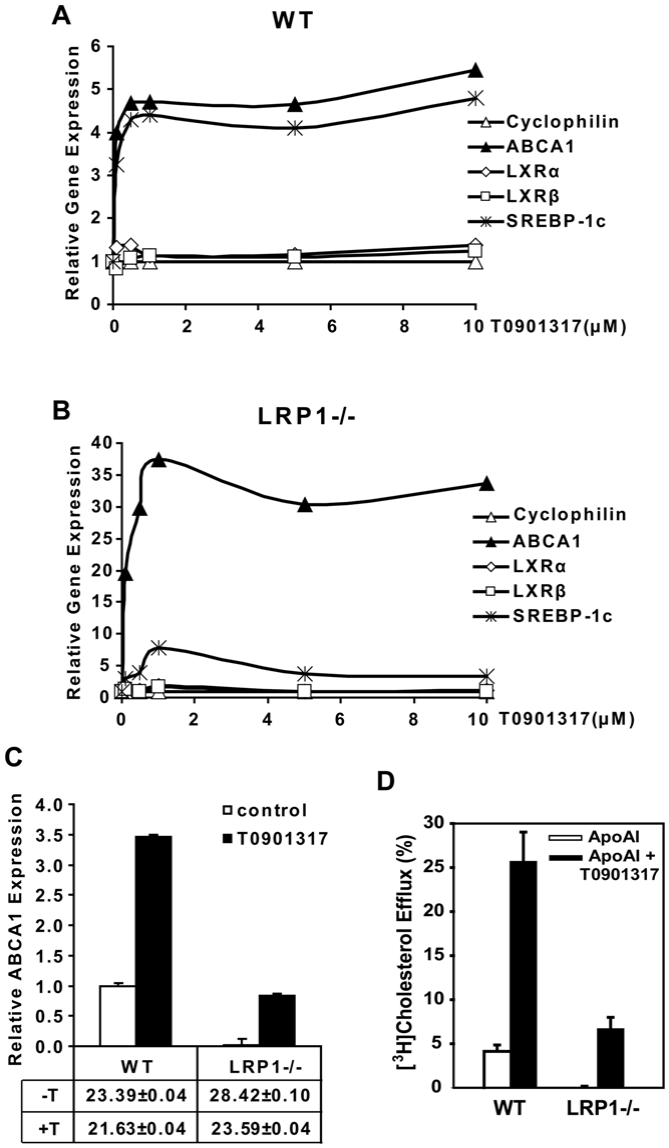
T0901317, a synthetic agonist of LXRs, up-regulates the expression of ABCA1. *A&B*. Concentration-response curves of T0901317. WT (A) and LRP1−/− (B) SMCs were treated with the indicated concentrations of T0901317 for 48 h and mRNA levels of ABCA1 and LXRs was subsequently determined by real-time PCR. Cyclophilin was used for normalization and SREBP-1c, a known LXR-regulatory gene [49], was added as a positive control. T0901317 up-regulated ABCA1 transcription at a low concentration of 0.1 µM and reached its maximum effect at approximately 1 µM in the absence or presence of LRP1. *C*. RT-PCR analysis of ABCA1 expression in WT and LRP−/− SMCs treated for 24 h with 5 µM T0901317. *C_T_* values in the absence (−T) and presence (+T) of the LXR agonist are shown±S.D. Treatment of LRP−/− SMCs with LXR agonist restored ABCA1 mRNA expression to wild type levels. Assays were performed in triplicate, standard deviations are shown. *D*. ApoAI-mediated cholesterol efflux was analyzed both in WT (white bars) and LRP1−/− (black bars) SMCs in the presence and absence of T0901317 using [^3^H]-labeled cholesterol. Radioactivity released from the cell into the medium was measured by liquid scintillation counting, and cellular lipids were extracted and analyzed for [^3^H]-sterol. Data are expressed as percentage of total (cell plus medium) [^3^H]-sterol released into the medium. A significant increase in apoAI-mediated cholesterol efflux was observed in both cell lines in the presence of T0901317. ApoAI-mediated cholesterol efflux was barely detectable in the LRP1−/− SMCs in the absence of prior T0901317 stimulation. The assay was performed in quadruplicate, and values are expressed as mean±S.D.

Like most other nuclear receptors that form heterodimers with RXRs, LXRs reside within the nucleus, bound to LXR response elements (LXREs) and complexed with corepressors. In the absence of ligand activation, these corepressors diminish the transcriptional activity of LXRs [27]. To explore whether the LXR agonist T0901317 [28] could activate the LXR/RXR complex, we added different amounts of T0901317 to the LRP1−/− SMCs and performed real-time PCR to follow the transcriptional regulation of ABCA1. SREBP-1c, another LXR target gene, was monitored for comparison. LXRα, LXRβ and cyclophilin are not regulated by LXRs and served as controls. As shown in the Figure 6B, T0901317 potently up-regulated ABCA1 and SREBP-1c transcription already at a low concentration of 0.1 µM in the LRP1-deficient SMCs and reached its maximal effect at about 1 µM. A similar dose-dependent response to T0901317 was seen in the WT SMCs (Figure 6A). However, the relative increase of ABCA1 mRNA expression was several fold greater in the LRP1−/− SMCs than in the wild type cells. Treatment with the LXR activator restored ABCA1 mRNA expression to wild type levels (Figure 6C). These data suggest that excessive repression of the LXR/RXR complex on the ABCA1 promoter, which can be overcome by treatment with an LXR agonist, may be responsible for its reduced transcription in cells lacking LRP1.

Because ABCA1 regulates the efflux of cellular cholesterol through direct interaction with ApoAI [29], [30], we measured ApoAI-mediated cholesterol efflux in the WT and LRP1−/− SMCs in the presence and absence of T0901317. As we expected, a diminished cholesterol efflux was observed in the absence of LRP1 due to the low level of ABCA1 expression. Cholesterol efflux increased significantly in the LRP-deficient cells following T0901317 stimulation along with increased expression of ABCA1 (Figure 6D).

### ABCA1 transcription is suppressed by LXR antagonists

**Figure 7:**
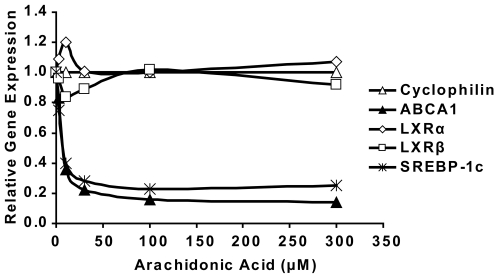
Arachidonic acid suppresses ABCA1 expression in WT SMCs. The indicated amount of arachidonic acid was administrated to WT SMCs to examine its effect on the expression of ABCA1 and LXRs. Cyclophilin was used for normalization and SREBP-1c, another LXR target gene, was measured as a positive control. Cells were incubated with the indicated amount of arachidonic acid for 6 h prior to real-time PCR. Arachidonic acid potently suppressed ABCA1 transcription at a low concentration of 3 µM and reached its maximum inhibitory effect at 100 µM.

Arachidonic acid can competitively antagonize T0901317-dependent activation of the LXR [31]. To examine its effects on the transcription of the LXR target genes ABCA1 and SREBP-1c, we administrated different amounts of arachidonic acid to wild type SMCs and analyzed ABCA1 transcription by real-time PCR. Our data show that arachidonic acid strongly suppresses ABCA1 transcription already at low concentrations of 3 µM and reaches its maximal inhibitory effect at 100 µM (Figure 7). Because the LRP1-deficient cells accumulate more cholesteryl esters and free fatty acids, this result suggest that excessive production of unsaturated fatty acids could be responsible for the inhibition of the LXR-dependent transcription in the absence of LRP1, leading to the observed reduction in ABCA1 expression. The increase in LXRα mRNA expression (Figure 5) may reflect a compensatory response by the cell to counter this increased inhibition.

### Loss of LRP1 function leads to cPLA_2_ hyper-phosphorylation

**Figure 8:**
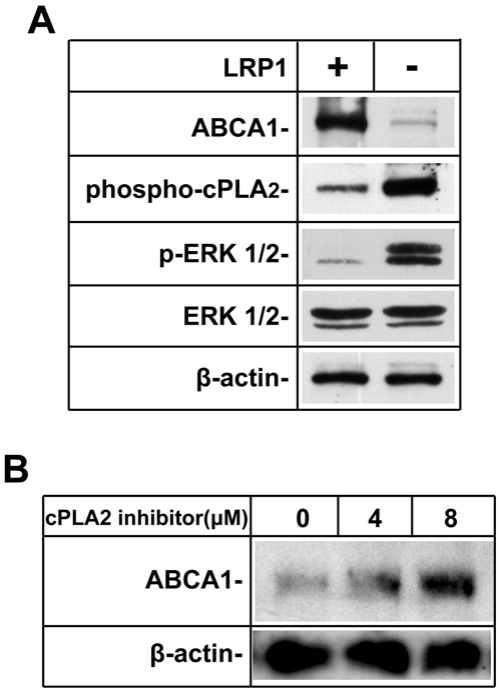
Phosphorylation of cPLA_2_ is markedly increased in LRP1-deficient SMCs. *A*. Western blotting was performed to determine the phosphorylation state of cPLA_2_ and ERK in the presence and absence of LRP1 in primary SMCsR A significant increase in cPLA_2_ and ERK phosphorylation was detected using rabbit anti-phospho-cPLA_2_ (Ser^505^) and anti-phospho-ERK antibodies. β-actin was used as a loading control. Five independent experiments were analyzed and quantitated. ABCA1 protein expression in LRP1−/− was 0.21±0.07, phosphorylated cPLA_2_ was increased by 2.73±0.35, and p-ERK1/2 was 3.37±0.56 compared to LRP+/+ cells. *B*. cPLA_2α_ inhibitor restores ABCA1 expression in LRP1-deficient SMCs. LRP1−/− SMCs were grown to confluence and treated with the indicated concentrations of the cPLA_2α_ inhibitor (Calbiochem, Cat. No. 525143) for 24 h. Dose-dependent increases in ABCA1 protein expression after administration of the cPLA_2α_ inhibitor were detected by Western blotting. β-actin was used as a loading control.

Extracellular regulated kinase (ERK) 1/2, also called mitogen-activated protein kinase (MAPK), has been reported to phosphorylate cytosolic phospholipase A2 (cPLA_2_) on Ser^505^, which induces the activation of this 85 kDa cellular enzyme [32], [33], [34]. Activated cPLA_2_ then translocates to membrane vesicles and selectively releases arachidonic acid from the *sn-2* position of membrane phospholipids [35]. Thus, increased cPLA_2_ activity results in more arachidonic acid production, and this could lead to excessive inhibition of LXR and reduced ABCA1 transcription. To investigate this possibility, we performed Western blotting using a specific anti-phospho-cPLA_2_ (Ser^505^) antibody. In the absence of LRP1, ERK is activated as a result of increased mitogenic, PDGFR-β mediated signaling [17], [18]. Significantly increased phosphorylation of cPLA_2_ was seen in the absence of LRP1 in response to increased ERK1/2 phosphorylation (Figure 8A), suggesting that excessive production of arachidonic acid may indeed be responsible for the reduced expression of ABCA1 in the absence of LRP1. To further test this hypothesis, we applied a cPLA_2α_ inhibitor to the LRP1-deficient SMCs and detected the expression of ABCA1 protein by Western blotting. Consistent with our hypothesis, ABCA1 protein levels where increased in a dose-dependent manner by this inhibitor (Figure 8B). This result shows that LRP1 controls cPLA_2_ activity, which in turn regulates the expression of ABCA1.

## Discussion

In the present study we have shown that LRP1 controls the expression of ABCA1, a major lipid transport protein in the plasma membrane that mediates cellular cholesterol export. Loss of LRP1 results in greatly reduced ABCA1 protein expression, which is caused by decreased LXR-mediated gene transcription. Increased mitogenic signaling in the absence of LRP1 activates ERK1/2 [17], [18], which in turn stimulates cPLA_2_ to release arachidonic acid, a potent LXR antagonist, from cellular phospholipids. This potent transcriptional repression of ABCA1 occurs in cultured cells (Figure 3) as well as in the vascular wall *in vivo* (Figure 2), where it results in a significant reduction of cholesterol export from LRP1-deficient smooth muscle cells (Figure 6D) and in the accumulation of excess cholesterol in the aortic wall (Figure 1). Reduced ABCA1 expression in the aorta of smLRP1−/− mice thus results in impaired cholesterol efflux and contributes to the excessive cholesterol accumulation *in vivo*. This mechanism may explain at least in part the greatly accelerated atherosclerosis that occurs in mice lacking LRP1 specifically in their smooth muscle cells. LRP1 thus functions as a physiological integrator of cellular lipid homeostasis with signals that regulate cellular proliferation and vascular wall integrity.

We observed increased accumulation of cholesterol and neutral lipids in the aortic wall and in LRP1-deficient smooth muscle cells (Figure 1 and 3). However, this cannot be explained entirely by increased lipid uptake from the circulation. First, LRP1 is a powerful lipoprotein uptake receptor, and loss of LRP1 expression thus should result in reduced, not increased cholesterol accumulation. On the other hand, we detected a minor compensatory increase in the level of LDLR expression (∼1.7 fold), consistent with earlier observations in the LRP1-deficient liver [36], which may contribute to the cholesterol elevation in LRP1 deficient aortas of LDLR-expressing mice. However, neither increased LDLR-mediated cholesterol uptake nor endogenous cholesterol biosynthesis can explain the massively increased cholesterol accumulation and atherosclerosis that occurs in smLRP- mice that also lack functional LDL receptors [18]. Circulating plasma lipoprotein levels are not altered by the presence or absence of LRP1 in SMCs [18], but are primarily determined by the presence or absence of the LDL receptor in the liver. Taken together, these findings suggested a functional defect in cellular cholesterol export from vascular SMCs as a potential underlying mechanism. We therefore investigated whether the expression of ABCA1, a major cholesterol transporter in the plasma membrane, might be altered in the absence of LRP1. As we had suspected, ABCA1 expression was greatly reduced in LRP1-deficient vessels as well as cultured primary smooth muscle cells. That ABCA1 does indeed have a significant atheroprotective role *in vivo* is further supported by a recent study from the Hayden laboratory [37], which reported tissue-specific roles of ABCA1 that influence atherosclerosis susceptibility.

What is the mechanism by which LRP1 controls cellular ABCA1 expression and cholesterol export? ABCA1 expression is tightly regulated at the transcriptional level, although post-transcriptional modulation has also been described [12], [25], [38], [39]. In this study, we have shown that ABCA1 is down-regulated at the transcriptional level in the absence of LRP1. Although the most important regulators of ABCA1 expression are the nuclear hormone receptors LXR and RXR [40], [41], [42] which form obligate heterodimers bound to LXREs within its promoter [26], [27], changes in the expression levels of LXRs and RXRs cannot explain the diminished ABCA1 expression in the absence of LRP1 (Figure 5), suggesting that the reduced ABCA1 expression is caused by transcriptional repression. LXR/RXR heterodimers reside in a complex with co-repressors. In the absence of activators, the transcriptional activity of ABCA1 is repressed [27]. Binding of activators to LXR/RXR heterodimers results in a conformational change and ABCA1 transcription [27]. In Figure 6 we have shown that a synthetic ligand of LXR, T0901317 [28], is able to activate the transcription of ABCA1, restore ABCA1 mRNA to wild type levels, and normalize ApoAI-mediated cholesterol efflux in the LRP1-deficient SMCs. These results suggested that in the absence of LRP1, although cells express normal amounts of LXRs and RXRs, the LXR/RXR heterodimers are bound to their co-repressors and are thus functionally repressed. This explains the greatly diminished basal expression of ABCA1 in the LRP1−/− cells. In the presence of the synthetic ligand, the LXR/RXR heterodimers are activated and readily induce ABCA1 transcription. The much lower baseline expression of ABCA1 in the LRP1-deficient SMCs further suggested, that an endogenous LXR antagonist may be responsible for this nearly complete transcriptional repression.

TLC analysis of total cellular lipid extracts from wild type and LRP1−/− cells showed that LRP1-deficient SMCs accumulate more free fatty acids compared to wild type cells. Polyunsaturated fatty acids are known to competitively antagonize activation of LXR by oxysterols and T0901317 [31]. To explore if an excess of polyunsaturated fatty acids could mediate the repression of LXR-dependent ABCA1 transcription in the absence of LRP1, we incubated wild type SMCs with increasing concentrations of arachidonic acid. The powerful reduction of ABCA1 and SREBP-1c transcription in the wild type cells by administration of exogenous arachidonic acid suggests that excessive accumulation of polyunsaturated fatty acids in the LRP1-deficient cells could indeed explain the observed reduction of ABCA1 expression in these cells.

As a polyunsaturated fatty acid, arachidonic acid is released from the *sn*-2 position of phospholipids by phospholipase A2 (PLA_2_). Mammalian cells contain several forms of PLA_2_ including secretory PLA_2_ (sPLA_2_), calcium-independent PLA_2_, and a cytosolic cPLA_2_. cPLA_2_ shares no homology with other PLA_2_ enzymes, and is the only well characterized PLA_2_ that preferentially hydrolyzes arachidonic acid from phospholipids at the *sn*-2 [34], [43]. In addition to being converted to potent inflammatory lipid mediators, which may independently contribute to the increased atherosclerosis of smLRP−/− mice by promoting macrophage recruitment, arachidonic acid is itself a key regulator of cellular signaling. The importance of arachidonic acid thus ensures that its levels are tightly controlled. As the crucial enzyme in mediating arachidonic acid release, cPLA_2_ is rapidly activated by increased concentrations of cytosolic Ca^2+^ and by serine phosphorylation [44]. cPLA_2_ contains a consensus sequence (Pro-Leu-Ser^505^-Pro) for phosphorylation by the MAPK ERK. ERK efficiently phosphorylates cPLA_2_ at Ser^505^, which increases its enzymatic activity [32]. Previous studies have shown that loss of LRP1 expression results in the elevated expression of PDGFRβ, which subsequently activates downstream ERK signaling [17], [18], [45]. Consistent with our previous findings [18], we observed increased phosphorylation of ERK1/2 in the absence of LRP1 (Figure 8), resulting in increased phosphorylation and activation of cPLA_2_, and thus accelerated phospholipid and arachidonic acid turnover. Administration of a cPLA_2_ inhibitor increased the expression of ABCA1 in the LRP-deficient cells, which further supports a mechanism by which over-production of polyunsaturated fatty acids, including arachidonic acid, is probably the underlying cause for the reduced ABCA1 expression in the absence of LRP1. Thus, suppression of ABCA1 transcription in the LRP1-deficient SMCs is likely due to repression of the LXR/RXR complexes by an endogenously produced antagonist and this is just one example for the altered LXR activity mediated by increased cPLA_2_ activity.

In summary, the present study has shown that LRP1 participates in apoAI-mediated efflux of cholesterol and phospholipids by controlling ABCA1 expression at the transcriptional level. These findings further emphasize the importance of LRP1 as an integrator of lipoprotein transport and cellular signals that regulate cell proliferation and migration, as well as cellular lipid homeostasis.

## Materials and Methods

### Materials

cPLA_2α_ inhibitor (N-{(2S,4R)-4-(Biphenyl-2-ylmethyl-isobutyl-amino)-1-[2-(2,4-difluorobenzoyl)-benzoyl]-pyrrolidin-2-ylmethyl}-3-[4-(2,4-dioxothiazolidin-5-ylidenemethyl)-phenyl]acrylamide, HCl) was from Calbiochem (Cat.No. 525143).

### Animals

*WT* and *smLRP1−/−* mice were maintained on a 129SvEv and C57BL/6J hybrid background by intercrossing of Sm22Cre+;LRP^flox/flox^ with hybrid LRP^flox/flox^ animals from the larger colony pool to prevent allele fixation. Sex and weight-matched littermates were used for all experiments. All mice were housed in an animal facility with 12h light/12h dark cycles. The animals were fed a standard rodent chow diet (Diet 7001, Harlan Teklad, Madison, WI) and water *ad libitum*. Male and female animals between 2 and 18 months of age were used throughout the studies. No sexual dimorphism of phenotype was observed. All procedures were performed in accordance with the protocols approved by the Institutional Committee for Use and Care of Laboratory Animals of the University of Texas Southwestern Medical Center at Dallas.

### Primary SMC culture

Primary mouse aortic SMCs were generated using the explant technique as previously described [46]. Briefly, aortas were dissected out under sterile conditions and rinsed twice with PBS. The connective tissue and adventitia were carefully removed. The aorta was opened longitudinally and the intima on the luminal surface was scraped off. The aorta was then cut into small pieces and transferred into a T25 flask containing high glucose (4.5 g/L) DMEM supplemented with 15% fetal calf serum (FCS), 100 U/ml penicillin and 100 mg/ml streptomycin. Outgrowing smooth muscle cells were detached by incubation with 0.25% trypsin-EDTA solution and cultured at 37°C in 5% CO_2_.

### Analysis of cholesterol levels in mouse aorta

Sterols in the mouse aorta were analyzed by gas chromatography and mass spectrometry (GC/MS) as previously described [47]. Briefly, the entire aorta from the aortic root to the iliac bifurcation was dissected out and the adventitia was removed. The aorta was weighed after superficial drying with tissue paper. Aortas were then dissolved in 1 ml of 0.1 M NaOH and vortexed for 30 min. An aliquot of ethanol containing the internal standards 5α-cholestane (50 µg) and epicoprostanol (2 µg) was added to 100 µl of tissue lysate, and sterols were hydrolyzed by heating to 100°C in 100 mM ethanolic KOH for 2 h. Lipids were extracted in petroleum ether, dried under nitrogen, and derivatized with hexamethyldisilazane-trimethylchlorosilane. GC/MS analysis was performed by using a 6890N gas chromatograph coupled to a 5973 mass selective detector (Agilent Technologies, Palo Alto, CA). The trimethylsilyl-derived sterols were separated on an HP-5MS 5%-phenyl methyl polysiloxane capillary column (30 m×0.25 mm inner diameter x 0.25 µm film) with carrier gas helium at the rate of 1 ml/min. The temperature program was 150°C for 2 min, followed by increasing the temperature by 20°C per min up to 280°C and holding it for 13 min. The injector was operated in the splitless mode and was kept at 280°C. The mass spectrometer was operated in selective ion monitoring mode. The extracted ions were 458.4 (cholesterol), 343.3 (desmosterol), 458.4 (lathosterol), 456.4 (zymosterol), 382.4 (campesterol), 393.4 (lanosterol), and 396.4 (β-sitosterol).

### Western blotting

Aortas or cell pellets were homogenized in lysis buffer (1% Triton X-100, 0.5% sodium deoxycholate, 0.1% SDS, 150 mM NaCl, 2 mM EDTA, 50 mM Tris-HCl, pH 7.5) containing freshly added proteinase inhibitors (P8430, Sigma) and phosphatase inhibitors (P2850 & P5726, Sigma). After centrifugation at 20,000 xg for 30 min at 4°C, the supernatant was used for Western blotting and the pellet was discarded. Protein extracts were separated on 4–15% SDS-PAGE gel and transferred to nitrocellulose membranes (HybondTM-C Extra, RPN303 E, Amersham Biosciences). Membranes were blocked with 5% skim milk, probed with an appropriate primary antibody (rat anti-mouse ABCA1: NB400-164, Novus; anti-phospho-cPLA_2_: 2831, Cell Signaling; anti-LDLR: 3143, Herz Lab; anti-LRP1: 377, Herz Lab), incubated with an appropriate horseradish peroxidase-conjugated secondary antibody (anti-rat IgG: NA9350; anti-rabbit IgG: NA934V; anti-mouse IgG: NA931V; Amersham Biosciences), and then developed with an enhanced chemiluminescence detection kit (RPN 2132, Amersham Biosciences).

### Immunofluorescence microscopy

Mice were perfusion-fixed via the left cardiac ventricle with warmed Hank's balanced salt solution-Hepes (20 mM, pH 7.3), followed by the same solution containing 4% (w/v) paraformaldehyde. The aorta was removed and divided into small pieces. Tissues were immersion-fixed for an additional hour followed by treatment with a mixture of 60% (v/v) methanol, 10% (v/v) glacial acetic acid, 30% (v/v) inhibisol (1,1,1-trichloroethane) for 24 h. Tissues were then embedded in paraffin. Paraffin sections (5 µm thick) were dewaxed in three changes of xylene (10 min each), and rehydrated into PBS (10 mM phosphate buffer, pH 7.2, 0.15 M NaCl). The sections were washed with 50 mM NH_4_Cl in PBS for 30 min and blocked by incubation for 1 h with TBS (10 mM Tris-HCl, pH 9.0, 150 mM NaCl) containing 10% (v/v) normal goat serum and 1% (w/v) bovine serum albumin (BSA). Samples were then incubated overnight at 4°C with a rat monoclonal antibody against mouse ABCA1 (NB400-164, Novus) at a 1∶20 dilution. Sections were washed three times in TBS containing 0.1% BSA, and the bound primary antibody was detected by incubation for 2 h with Alexa-Fluor 568-conjugated goat anti-rat IgG (10 µg/ml, Molecular Probes, Eugene, OR). The tissue slides were then washed three times in TBS containing 0.1% BSA, rinsed with water, and mounted on a coverslip with Fluorescence Mounting Medium (DakoCytomation). Images were taken using Leica TCS SP confocal microscope.

### Oil Red O staining

Cells were grown on coverslips for two days and then fixed with 10% formalin in phosphate-buffered saline for 1 h at room temperature. After washing three times with deionized water, cells were stained with Oil Red O in isopropyl alcohol at a concentration of 4 mg/ml. Finally, each coverslip was washed for 10 min with deionized water and mounted on glass slides for microscopic evaluation.

### Lipid analysis by thin layer chromatography (TLC)

SMCs were harvested and lysed in buffer B (250 mM sucrose, 100 mM KCl, 50 mM Tris-HCl, pH 7.4) after 4 h incubation with ^14^C-labelled oleic acid. Part of the cell lysate was utilized for protein measurement. Cell lysate containing equal amounts of protein was further processed for lipid extraction by 500 µl of chloroform/methanol (2∶1, v∶v). Lipid extracts were then dried and dissolved in 130 µl of chloroform/methanol (1∶1, v∶v). 60 µl of the lipid extracts were loaded (30 µl/lane, duplicate) onto a 20×20 cm silica gel plate (805013, POLYGRAM SIL G, MACHEREY-NAGEL) for cholesterol ester, triglyceride, and free fatty acid separation by TLC. 20 µl of ^3^H-labelled cholesterol recovery solution containing cholesterol oleate ester (30 µg), triglyceride (10 µg) and oleate (20 µg) was used as lipid standards. Cholesteryl ester, triglyceride, and free fatty acid separation was performed using hexane/ethyl ether/acetic acid (80∶20∶1, v∶v∶v) as the solvent. TLC plates were dried in a hood, and exposed to a phosphor imaging plate for 24 h at room temperature. The imaging plate was analyzed by the Storm 820 Phosphor imager (Molecular Dynamics, Sunnyvale, CA).

### Quantitative real-time PCR

Total RNA from SMCs was prepared using RNA STAT-60 from Tel-Test Inc. Equal amounts of RNA were treated with RNAase-free DNAase I (Ambion Inc.). First-strand cDNA was synthesized from 2 µg of DNAase I–treated total RNA with random hexamer primers using TaqMan Reverse Transcription Reagents (N808-0234, Applied Biosystems). Specific primers for each gene were designed using Primer Express software (Applied Biosystems). The real-time PCR reaction was set up in a final volume of 20 µl containing 20 ng/µl cDNA, 2.5 µM forward and reverse primers, and 10 µl of 2x SYBR Green PCR Master Mix (4312704, Applied Biosystems). PCR reactions were carried out in a 384-well plate using the ABI PRISM 7900HT Sequence Detection System (Applied Biosystems). All reactions were done in triplicate. The relative amount of mRNA was calculated using the comparative threshold cycle (ΔΔ*C_T_*) method as recommended by the manufacturer in the Applied Biosystems protocols. The primers for real-time PCR were listed as follows: mouse ABCA1, 5′-CGTTTCCGGGAAGTGTCCTA-3′ (forward), 5′-GCTAGAGATGACAAGGAGGATGGA-3′ (reverse); mouse LXRα, 5′-TCTGGAGACGTCACGGAGGTA-3′ (forward), 5′-CCCGGTTGTAACTGAAGTCCTT-3′ (reverse); mouse LXRβ, 5′-CTCCCACCCACGCTTACAC-3′ (forward), 5′-GCCCTAACCTCTCTCCACTCA-3′ (reverse); mouse RXRα, 5′-CAGTACGCAAAGACCTGACCTACA-3′ (forward), 5′-GTTCCGCTGTCTCTTGTCGAT-3′ (reverse); mouse RXRβ, 5′-AAGTGTCTGGAGCACCTGTTCTT-3′ (forward), 5′-CTCCATGAGGAAGGTGTCAATG-3′ (reverse); mouse SREBP-1c, 5′-GGAGCCATGGATTGCACATT-3′ (forward), 5′-GGCCCGGGAAGTCACTGT-3′ (reverse); mouse cyclophilin, 5′-TGGAGAGCACCAAGACAGACA-3′ (forward), 5′-TGCCGGAGTCGACAATGAT-3′ (reverse). Mouse cyclophilin mRNA was used as the invariant control.

### ApoAI-mediated cholesterol efflux assay

ApoAI-mediated cholesterol efflux was analyzed as previously described [48]. Briefly, SMCs were seeded into 16 mm wells and radiolabeled starting at 60% confluence in high glucose (4.5 g/L) DMEM containing 10% FCS and 0.3 µCi/ml [^3^H]-cholesterol (C8794, Sigma). Confluent cells were then loaded with 30 µg/ml unlabeled non-lipoprotein cholesterol for 24 h, and equilibrated for 24 h prior to 24 h incubation of 10 µg/ml apoAI. Equilibration and apoAI administration were performed in the presence or absence of 10 µM T0901317. At the end of the incubation, media were collected and centrifuged at 2,000 xg for 10 min to remove cell debris. Radioactivity in the medium was measured by liquid scintillation counting (LS-60001C, Beckman Instruments Inc.). Cellular lipids were extracted and analyzed for [^3^H]-sterol. This assay was performed in quadruplicate. Data are expressed as percentage of total (cell plus medium) [^3^H]-sterol appearing in the medium. Values are expressed as mean±S.D.

### Statistics

Unpaired two-tail Student t-test was used for statistical analyses. A *p* value < 0.05 was considered significant.

## Supporting Information

Figure S1 GC/MS analysis of cholesterol in the mouse aorta. The aorta from the aortic root to the iliac bifurcation was isolated, and the surrounding connective tissue was carefully removed under a dissecting microscope. The aorta was weighed after superficial drying with tissue paper. Cholesterol in the aorta was determined by GC/MS and the amount of total cholesterol (µg) was normalized by the weight of the aorta (g). Increased free and esterified cholesterol levels were found in the aorta of 18 month old smLRP1−/− mice (compared to wildtype mice of the same age, n = 4/group).
(0.19 MB TIF)
